# Identification of Novel QTLs Associated with Frost Tolerance in Winter Wheat (*Triticum aestivum* L.)

**DOI:** 10.3390/plants12081641

**Published:** 2023-04-13

**Authors:** Parisa Bolouri, Kamil Haliloğlu, Seyyed Abolghasem Mohammadi, Aras Türkoğlu, Emre İlhan, Gniewko Niedbała, Piotr Szulc, Mohsen Niazian

**Affiliations:** 1 Department of Field Crops, Faculty of Agriculture, Ataturk University, 25240 Erzurum, Turkey; 2 Department of Plant Breeding and Biotechnology, Faculty of Agriculture, University of Tabriz, Tabriz 5166616471, Iran; 3 Department of Field Crops, Faculty of Agriculture, Necmettin Erbakan University, 42310 Konya, Turkey; 4 Department of Molecular Biology and Genetics, Erzurum Technical University, 25240 Erzurum, Turkey; 5 Department of Biosystems Engineering, Faculty of Environmental and Mechanical Engineering, Poznań University of Life Sciences, Wojska Polskiego 50, 60-627 Poznań, Poland; 6 Department of Agronomy, Poznań University of Life Sciences, Dojazd 11, 60-632 Poznań, Poland; 7 Field and Horticultural Crops Research Department, Kurdistan Agricultural and Natural Resources Research and Education Center, Agricultural Research, Education and Extension Organization (AREEO), Sanandaj 6616936311, Iran

**Keywords:** winter wheat, frost tolerance, LT_50_, chromosome 4A, SSR marker, yield-related traits

## Abstract

Low temperature (cold) and freezing stress is a major problem during winter wheat growth. Low temperature tolerance (LT) is an important agronomic trait in winter wheat and determines the plants’ ability to cope with below-freezing temperatures; thus, the development of cold-tolerant cultivars has become a major goal of breeding in various regions of the world. In this study, we sought to identify quantitative trait loci (QTL) using molecular markers related to freezing tolerance in winter. Thirty-four polymorphic markers among 425 SSR markers were obtained for the population, including 180 inbred lines of F_12_ generation wheat, derived from crosses (Norstar × Zagros) after testing with parents. LT_50_ is used as an effective selection criterion for identifying frost-tolerance genotypes. The progeny of individual F_12_ plants were used to evaluate LT50. Several QTLs related to wheat yield, including heading time period, 1000-seed weight, and number of surviving plants after overwintering, were identified. Single-marker analysis illustrated that four SSR markers with a total of 25% phenotypic variance determination were linked to LT50. Related QTLs were located on chromosomes 4A, 2B, and 3B. Common QTLs identified in two cropping seasons based on agronomical traits were two QTLs for heading time period, one QTL for 1000-seed weight, and six QTLs for number of surviving plants after overwintering. The four markers identified linked to LT_50_ significantly affected both LT_50_ and yield-related traits simultaneously. This is the first report to identify a major-effect QTL related to frost tolerance on chromosome 4A by the marker XGWM160. It is possible that some QTLs are closely related to pleiotropic effects that control two or more traits simultaneously, and this feature can be used as a factor to select frost-resistant lines in plant breeding programs.

## 1. Introduction

Winter wheat (*Triticum aestivum* L., 2n = 6x = 42, AABBDD) is a naturally formed allohexaploid species with seven groups of homoeologous chromosomes [1]. In Turkey, 49.37% of the total seed cultivated is wheat. As seed farming is relatively easy and suitable for mechanization, farmers often choose to cultivate these crops. According to TUIK data, Turkey’s wheat cultivation area constitutes 3.2% of the world wheat cultivation area as of the 2019–2020 production season [2]. Temperature is an important environmental factor that affects wheat production [1]. Cold temperatures and frost are fundamental non-biological factors that reduce wheat production worldwide. Cold tolerance is a complex trait in wheat and includes morphological, physiological, biological, and hereditary elements [3]. Such stressors are frequent during the life of the plant and can reduce the yield of agricultural products [4,5].

The most important stage of plant development is the flowering induction stage, which occurs in many plant species based on the response to seasonal changes caused by the surrounding environment. The mechanism of cold tolerance in winter wheat has a fundamental relationship with the need for vernalization, which causes a delay in the transition from the vegetative to the reproductive stage; the result is cold tolerance [5].

Abiotic stresses such as frost stress are complex quantitative traits, where numerous stress-responsive genes take part to ensure the survival of plants [4]. It is possible that in a wide range of plant species, such responses are controlled by quantitative trait loci (QTL) [6]. The ability of plants to survive frost temperatures is critical for long-term survival. Although field survival is the ultimate measure of winter hardiness of a variety, field survival as a selection tool is unreliable because of variable levels of winter severity on different winter crops [7]. Recently, analyses of plants grown in fields and natural environments have revealed that most frost-responsive genes detected in the laboratory are also responsive to frost stress in these environments [8].

LT_50_, the temperature predicted to be fatal to 50% of the plants, is typically used to quantify the frost tolerance of winter wheat cultivars. The way in which LT_50_ is measured varies among researchers [9]. Livingston [10] studied frost tolerance in different plant species (rye, wheat, barley, oat) and tested the plants after freezing. For this purpose, the frost-tolerance genotypes of barley and oats were subjected to −4, −7, −9, and −12 °C, and the more tolerant genotypes to −14, −16, and −18 °C. Plants were kept at each temperature for 2 h. Mahfoozi et al. [11] illustrated that the level of expression as well as the number of expressed proteins in plants grown in field conditions that experience cold acclimatization periods at sub-zero temperatures show much higher tolerance than plants acclimated to cold at low but above-zero temperatures (in controlled conditions).

To study different winter wheat cultivars, several biochemical, physiological, and morphological traits, such as plant height, heading time period, nutrient content, sucrose, glucose, raffinose, total sugar content, and the relationship between frost resistance genes, were investigated [12]. Studies have been conducted on protein factors such as transcription factors and protein kinases, which play a role in stress response and further regulation of gene expression [13]. Moreover, phenological, molecular, and metabolic analyses during vernalization have shown that there is a close relationship between the completion time of vernalization, the reduction of accumulation of metabolites, and the expression of frost-induced proteins [14].

The relationship between cold adaptation (acclimation) and other physiological events is important as cereals become tolerant to colder temperatures as a result of acclimatizing to cold exposure. In addition, adaptation activates different gene expression pathways [15]. Cold-activated genes may also be present in more than one gene locus. Response of plants to abiotic stress occurs via signal transduction [16]. Hannah et al. [17] reported that cold adaptation is the result of an increase or decrease in the expression of many genes. Thus, the controlling factor for frost tolerance depends on genetic (evolutionary) changes between vegetative and reproductive growth periods [18]. Therefore, the reaction of plants to low temperatures is primarily through activating metabolic pathways, followed by cell recognition (detection) and activation of genes that react to frost stress [19].

Wheat has both winter and spring growth phases, which are determined via vernalization (VRN) genes [20]. The genes involved in vernalization are located on the fifth chromosome and include homologous Vrn-A1, Vrn-A2, and Vrn-A3 genes [21]. The dominant or recessive alleles per locus lead to tolerance to frost or low temperatures in wheat with a winter growth type [18]. Freezing tolerance (Fr), which is linked to Vrn alleles, is an effective factor in gene stability. Fr-A_11_, also known as Fr loci, are located on group 5 chromosomes in the loci of Fr-B_1_ and Fr-D_1_ [22,23]. Another locus, Fr-A_2_, is located approximately 30 to 40 centimorgan (cM) to the vernalization locus Vrn-A_1_ [24,25] and is also located on the fifth chromosome of *T. monococcum* [26]. According to Baga et al. [24] and Börner et al. [27], genetic mapping studies have shown which Fr and VRN genes are autonomous loci, and these loci are the main sources of variations observed in freezing tolerance [21,23]. Recent research has shown that allelic variations in the Vrn-A_1_ locus significantly affect freezing tolerance. This QTL region associated with Fr-A_1_ for freezing tolerance is due to the pleiotropic effect of Vrn-A_1_ rather than being entirely dependent on the Fr-A_1_ gene [19,28] and is located on the group 5 chromosomes [18]. Shindo et al. [29] also observed the importance of multiple locations on chromosome 2B, which controls heading time in wheat. In addition to the importance of chromosomes of the B genome in terms of frost tolerance, several studies related to the association mapping of wheat indicated that there were significant relationships between genetic markers and other agronomic traits, such as heading time, on the chromosomes of the B genome. Chromosome 4A is significantly associated with plant height, heading time, number of seeds per spike, spike length, spikelet number, and 1000-seed weight [24,30,31].

The selection of complex genetic traits, such as frost tolerance, can be simplified in plant breeding programs when associated markers are identified [32]. QTL and DNA markers, which are complex properties, can be used for indirect selection in selection programs with the aid of a marker [33]. Most phenotypes are quantitative in nature, and thus, significant variation for a trait of interest may be assigned to one or more loci (QTL). Identification and validation of QTLs requires associating them with one or more molecular markers. Knowing the location and the number of loci-wrapped traits, such as frost tolerance, increases the efficiency of selection of such agronomic traits. In recent years, molecular markers have been used as a useful complement to classical breeding techniques in the selection of quantitative traits, such as freezing tolerance in wheat. The aim of the present study is to identify gene or gene regions (QTLs) related to frost tolerance by SSR markers. QTLs linked to frost tolerance traits can be used in plant breeding programs for winter wheat.

## 2. Results

### 2.1. Evaluation of Yield-Related Traits

The mean time period from planting to 50% heading time in Zagros and Norstar was 181 and 195 days, respectively. For the F_12_ population, it was 185 days. The mean 1000-seed weight in Zagros and Norstar was 40.34 and 40.73 g, respectively. For the F_12_ population, the mean was 40.16 ± 0.28 g. The number of surviving plants after overwintering in Zagros and Norstar was 37.25 and 46.75 seedlings, respectively. The mean number of surviving plants after overwintering for the F_12_ population was 44.85 seedlings. Statistically significant correlations between frost tolerance (LT_50_) were observed with heading time period (r = 0.154 *) and number of surviving plants after winter (r = 0.66). In addition, significant negative correlations were observed between winter survival and heading time period (r = −0.13) and 1000-seed weight (r = −0.223) (Table 1).

### 2.2. Evaluation of LT_50_ Values

The F_12_ population and their parents were examined using the freezing test, and LT_50_ values of the population varied in thermal range of −3 °C to −25 °C (ST1). The LT_50_ values of Zagros (Z) and Norstar (N) parents were determined as −4.5 °C and −24.34 °C, respectively. The Norstar variety showed maximum frost tolerance, with an LT_50_ value at −24.34 °C. The mean LT_50_ value for the F_12_ population showed a distribution close to the frost-sensitive Zagros parent; the mean LT_50_ value for the F_12_ population was −8.94 °C (Table 2) and was considered as the frost tolerance standard. In general, in the RIL population, 82 (45.5%) lines showed high tolerance, and 98 lines (54.44%) were susceptible to frost.

### 2.3. Molecular Evaluation (Single Marker Analysis)

Thirty-four SSR markers were identified to be polymorphic between parents and thus were used for single-marker analysis [34]. Four QTL regions were found for the LT_50_ value (Table 3). The QTL regions were associated with XBarc101, XGWM340, XGWM160, and XGWM493 markers. In terms of variation related to the LT_50_ value, XGWM160 had the highest phenotypic variation (12%), located on chromosome 4A and linked to LT_50_. The other three QTL regions expressed phenotypic variation from 2% to 7% (Table 3). These QTL regions were also located on chromosomes 2B and 3B, which were linked to LT_50_. Additionally, QTL analysis (SMA) for yield-related traits showed that two marker loci (XGWM413 and XGWM165) were located on 1B and 5AL-12-~10 chromosomes, respectively, which were related to the heading time period. One marker locus (XGWM160) on 4A was associated with the 1000-seed weight. Six marker loci (XGWM340 on 3B, XBarc 154 on 7A, XBarc100 on 5AL-12-~10, XGWM501 on 2B, XGWM160 on 4A, and XWMC765 on 5D chromosomes) were associated with number of surviving plants after overwintering in the F_12_ population (Table 4).

## 3. Discussion

Considering that low-temperature stress in late spring is a serious threat to winter wheat production, frost tolerance is one of the most important traits for wheat breeding programs. This trait is complex and controlled by QTLs. Although challenging, identification of these QTLs will greatly benefit agricultural development. Therefore, considering the importance of yield-related traits in winter wheat, such as heading time period, 1000-seed weight, and number of surviving plants after overwintering, as well as their effect on yield, many studies have focused on identifying the QTLs associated with these traits and characterizing the molecular control of these traits and their role in frost tolerance.

Different organs of winter wheat are different in terms of resistance to low-temperature stress, among which the leaves are the most sensitive to low-temperature stress. Although low-temperature stress during elongation and booting stages causes great damage to the young ear, reduces the number of distinct florets, and accelerates the degeneration of florets, it seems that genotypes with a longer heading time period are less damaged by frost stress. Because the biomass transferred to the sink organ decreases less in these genotypes, as a result, it does not reduce the grain yield [32,35,36,37,38]. Our studies revealed that there is a significant correlation between frost tolerance (LT50) and heading time period in the F12 population (r = 0.154 *). However, significant positive correlations (*p* < 0.05) were exhibited between heading time period and LT50, indicating a high response to selection of these traits (Table 1). Therefore, lines with a larger heading time period were also more tolerant to low temperatures and frost. Several QTLs with pleiotropic effects are involved in controlling this trait. There may be genetic linkage between frost tolerance and late maturity (winter growth habit) [32,35,36,37,38]. Our results indicate that with an increasing period of heading time, the expression of genes related to frost tolerance strongly increases. Heo et al. [39] reported that transcription factors are better expressed with extended periods of heading time and could be promising candidates for identifying the molecular mechanisms and fitness of freezing tolerance. In addition, the shortening of the day length in autumn leads to the induction of the FT1/VRN3 gene upstream of the key gene of springing VRN1 [40]. Downregulation of Cor/Lea (cold-responsive or cold-regulated/Late-embryogenesis-abundant) genes and frost tolerance under controlled conditions was reported by Fowler et al. [9]. There is strong evidence that the Cor/Lea gene can contribute to freezing tolerance [41].

Low temperature (LT) represents a critical environmental factor in determining winter survival (WS) of winter wheat species. This means that during the acclimation process, highly tolerant varieties accumulate dehydrin proteins and transcripts earlier and at a higher level than less tolerant varieties.

We identified four QTL linked to LT_50_ that controlled frost tolerance on three wheat chromosomes (B3, 2BL, and 4A), which described a total of 25% of phenotypic variation for LT50. In addition, a QTL identified on chromosome 4A had a major effect on LT_50_, and this accounted for 12% of the total phenotypic variance. For this purpose, it is possible to use QTLs with major effects linked to LT_50_ with a *p*-Value close to zero for selection in breeding programs. The segregation ratio of the percentage of tolerant F12 lines in the population shows that the resistance may be conditioned by more than one gene. Baga et al. [24] reported the importance of QTL regions on chromosomes 1D, 2A, 2B, 6D, and 7B in frost tolerance. However, the results of other studies indicate the existence of QTLs linked to frost resistance on chromosomes 5B, 5D, 5A, 2D, 2A, and 4B [40,42,43]. A previous study conducted by Sutka [44] emphasized the importance of QTLs linked to frost tolerance on chromosome 2B, which is consistent with our results. Traits related to yield, such as the number of surviving plants and frost resistance, were identified in chromosome 2B in our results. On the other hand, Kruse et al. [45] identified one QTL on chromosomes 5A and 4B associated with freezing tolerance by using 155 recombinant inbred lines with 663 molecular markers in F_2:5_ lines in bread wheat. Numerous studies have indicated the location of the freezing tolerance genes mapped to homologous 5th group chromosomes [23,32,44], showing the importance of chromosome 5A in frost tolerance [22,32,44,46]. Previously, the study by Ballesta et al. [47] found that at least one of the 175 SNP markers was related to the drought tolerance index, which explained up to 6% of the phenotypic changes. Forty-five SNPs were associated with more than one tolerance index (up to four agronomic traits). Most linkages were located on chromosome 4A, supporting the hypothesis that this chromosome plays a key role in drought tolerance and should be used for wheat improvement. In the present study, our results show for first time that among QTLs linked to frost tolerance, a major effect QTL (12%) was identified with the aid of GWM 160 SSR marker on chromosome 4A, which indicates the importance of this locus on chromosome 4A. QTL have been verified by genome-wide association studies using a diverse panel of 276 winter wheat genotypes of one QTL on chromosomes 3A, 3D, 4A, and 7D [48]. Although Galiba et al. [49] and Juhasz et al. [50] emphasized that genes controlling osmotic regulation and proline content are mainly located on chromosomes 5D and 5A, the contribution of other chromosomes, especially 4A in frost tolerance, cannot be ignored. Based on our results, a major QTL on chromosome 4A is linked with both frost resistance and number of surviving plants after overwintering. This indicates the importance of chromosome 4A in frost tolerance in winter wheat. Single marker analysis was preferred due to its simplicity and specific conditions to determine QTLs.

## 4. Materials and Methods

### 4.1. Plant Material and Mapping Population

The Norstar variety is a cold-tolerant (LT_50_ −22.25 °C) variety developed in Saskatchewan, Canada, in the 1980s and was used as the maternal parent. The other parent, Zagros, is an early and summer wheat variety that was developed by the International Agricultural Research Center for Dry Areas (ICARDA) as highly sensitive to cold (LT_50_ −3.5 °C) [36]. The mapping population consisted of 182 recombinant inbred lines (RILs) (F_12_), derived from a cross between Norstar and Zagros; two parental lines were used as genetic material.

The F_12_ generation seeds were planted in a greenhouse with temperature conditions of 20 °C and a 10/14 h (D/N) photoperiod. After 5 weeks, when the growing seedlings reached the 3- to 4-leaf stage [51], the plants were transplanted to a freezing chamber for vernalizing at 2 ± 0.5 °C for 6 weeks. The vernalized seedlings were then prepared for a freezing test.

### 4.2. Freezing Tolerance Screening

**Figure 1:**
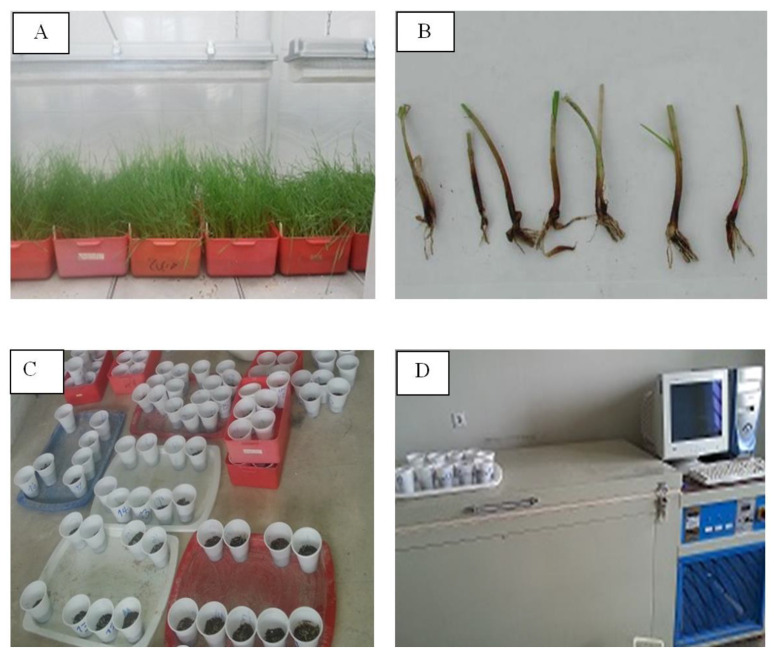
Stages of freezing test; (**A**) Cultivation of genotypes in the greenhouse, (**B**) Preparing seedlings for frost exposure (freezing test), (**C**) Preparing seedlings for transfer to the freezing test machine, (**D**) Freezing test machine.

Freezing tolerance was evaluated from −3 °C to −25 °C at −2 °C increments in 12 test ranges after cold acclimation according to Fowler et al. [12] and Mahfoozi et al. [51] (Figure 1) (ST1). Vernalized seedlings of the genotypes were placed in plastic pots and wetted and kept at 2–4 °C for 2 days for the adaptation assay freezing test. At the end of this period, seedlings were transferred to a programmable freezer for array freezing tests (Figure 1C,D) [32,52] and kept for 1 h at each temperature point. After the freezing test stage, samples (pots) were kept in a growth chamber with a 10/14 h (D/N) photoperiod at 4 °C for 24 h. Samples were then allowed to regrow in the greenhouse with a 10/14 h (D/N) photoperiod at 20 °C for 3 weeks, and LT_50_ was recorded for the entire population. The final LT_50_ was calculated after probity transformation. Accordingly, if at least 5 out of 10 plants survived, the degree of frost tolerance was considered for that genotype (LT_50_ value).

### 4.3. Field Experiment

The F_12_ RILs were planted at the Research Farm of Ataturk University for 2 years (2014–2016) in a randomized complete design (RCBD) with two replications. Thereafter, the following yield-related parameters were determined: heading time, time period between the sowing date and the time when almost half of the spikes of each row of plants emerged from the flag leaf sheath, 1000-seed weight, number of surviving plants after winter, number of plants that emerged after winter. This experiment was performed to identify any probable correlation between these traits and LT_50_ and any possible common QTL governing both traits.

### 4.4. Genotyping

Nuclear DNA was extracted from young leaves of wheat plants of individual F_12_ ten-day seedlings germinated from seeds of each genotype, as previously described [53]. DNA samples were examined with 0.8% agarose gel electrophoresis for quality and then quantified by a Nanodrop device. DNA samples were diluted to 20 ng/µL concentration. A set of 425 SSRs from the Wheat database (XBARC, XCFA, XCFD, XGWM, XWMC, and XWMS) involve the 21 chromosomes of wheat. Wheat microsatellites (SSR) were chosen from http://www.graingenes.org (accessed on 5 January 2022). For molecular analysis, parental-line polymorphisms were assessed by 425 SSR primer pairs distributed on all wheat chromosomes (ST2). Thirty-four polymorphic SSR primer pairs were used for genotyping the F_12_ population. QTL mapping was performed based on single mapping. PCR was performed to amplify the sequence in the SSR molecular markers (94 °C for 1 min (one cycle); 94 °C for 20 s, 50–62 °C for 35 s, 72 °C for 45 s (35–38 cycles), and final extension at 72 °C for 45 s (one cycle, then hold at 4 °C indefinitely) [54]. The PCR products were then loaded onto 6% polyacrylamide gels. Bands were separated by electrophoresis at 100 V containing 0.5 μg/mL ethidium bromide for approximately 2 h using 0.5 × TBE buffer, along with a DNA ladder, and examined under ultraviolet light. Finally, the gels were photographed using a digital camera (Model Nikon Coolpix500, Nikon, Japan) under UV light [55,56].

### 4.5. Data Analysis

Before analyzing the obtained phenotypic data, a normality test was performed with the SPSS program and the *Shapiro–Wilk* method was used for non-parametric analysis. The SAS program (SAS Institute, Inc, NC, USA. http://www.sas.com (accessed on 5 January 2022)) was used for variation analysis. LT_50_ values were determined by probit regression analysis from the SAS program [57]. QTL analysis was performed to identify the QTL associated with frost tolerance in winter wheat using 182 plants of an F_12_ population derived from crosses between two bread wheat genotypes using the MAPMANAGER-QTX20 program based on the values of Single Marker Analysis (SMA) [32]. The percentage of phenotypic variation explained by markers was calculated based on R-square regression analysis based on SMA using MAPMANAGER-QTX20 software [32]. Due to fewer polymorphic markers, no maps were constructed, and the QTL analysis used the SMA method.

## 5. Conclusions

Wheat culture is strongly affected by types of stress, such as cold and freezing. Therefore, the generation of cold-tolerant cultivars is one of the essential challenges to genetics and breeders. SSR markers, due to high efficiency, are useful for the detection of QTLs related to abiotic stress, such as cold. In this study, some primers were also linked to yield-related traits, such as number of plants surviving after overwintering, 1000-seed weight in field conditions, and frost tolerance, which suggests pleiotropic effects. Therefore, in a genotype with greater 1000-seed weight and a greater number of plants surviving after overwintering, the activity of frost tolerance genes is prolonged, and the expression of these structural genes is increased. In addition, QTL-rich regions on chromosome 4A were detected, supporting the hypothesis that this chromosome has a key role to play in frost tolerance and should be exploited for wheat improvement. In addition, the traits LT50, 1000-seed weight, and number of surviving plants after winter were located in the 4A genome and have been associated with frost tolerance. This suggests that a set of gene loci on a set of wheat chromosomes plays a role in the degree of frost tolerance. Other SSR markers and gene expression mechanisms should be investigated.

## Figures and Tables

**Table 1:** Correlation between studied agronomic traits.

	Heading Time Period (HT)	1000-Seed Weight (1000—SW)	Number of Surviving Plants after Winter (WS)	LT_50_
HT	1			
1000—SW	−0.097	1		
WS	−0.130	−0.223 **^1^	1	
LT_50_	0.154 *^2^	0.084	0.66 *	1

^1,2^ Significant at ** *p* = 0.01 and * *p* = 0.05 levels, respectively.

**Table 2:** The mean LT_50_ values in the recombinant lines (RILs) derived from Norstar × Zagros wheat cross.

LT_50_ (°C)	−1.5	−4.5	−7.5	−10.5	−13.5	−16.5	−19.5	−24.34
Genotype number	5	51	43	40	25	15	2	1

^1,2^ Significant at ** *p* = 0.01 and * *p* = 0.05 levels, respectively.

**Table 3:** QTL analyses of SSR markers for LT_50_ in the recombinant lines (RILs) derived from Norstar × Zagros wheat cross.

Characteristic	Marker	Location	* %PV	% *p*-Value
LT_50_	XBarc 101	2BL	7	0.002
	XGWM340	3B	2	0.036
	XGWM160	4A	12	0.000
	XGWM493	3B	4	0.004

* % PV: Phenotypic Variation.

**Table 4:** QTL analyses for the number of yield-related traits in the recombinant lines (RILs) derived from Norstar × Zagros wheat cross.

Characteristic	Marker	Location	% Phenotypic Variation (PV)	% *p*-Value
Heading time period	XBarc165 XGWM413	5AL-12~10 1B	2 6	0.032 0.000
1000-seed weight	XGWM160	4A	16	0.000
Number of surviving plants after winter	XBarc 154 XBarc100 XGWM501 XGWM340 XGWM160 XWMC765	7A, D 5AL-12~1 2B 3B 4A 5D	5 2 3 9 12 5	0.011 0.033 0.022 0.000 0.000 0.003

## Data Availability

Data are contained within the article and Supplementary Materials.

## Supplementary Materials

The following supporting information can be downloaded at: https://www.mdpi.com/article/10.3390/plants12081641/s1, Table S1: Freezing test LT_50_ values. Table S2: 425- SSR primers for QTL analysis among RIL accessions.
