# Supplementary material for: Identification of Novel QTLs Associated with Frost Tolerance in Winter Wheat (Triticum aestivum L.)
Source: Plants (Basel). 2023 Apr 13;12(8):1641. doi: 10.3390/plants12081641 (PMC10146367; doi:10.3390/plants12081641)
Supplement: Supplementary file 1 [file plants-12-01641-s001.zip › plants-2247413-supplementary S1.pdf]

Table S1. Freezing test LT$_{50}$ values.

LT$_{50}$	-3	-5	-7	-9	-11	-13	-15	-17	-19	-21	-23	-25
1	5/5	5/5	5/5	4/5	3/5	2/5						
2	5/5	5/5	3/5									
3	5/5	5/5	5/5	5/5	2/5	2/5						
4	5/5	5/5	5/5	5/5	4/5	4/5	3/5		2/5		2/5	
5	5/5	5/5	5/5	5/5	4/5	5/5	5/5					
6	5/5	5/5	5/5	5/5	4/5	5/5	5/5					
7	5/5	5/5	5/5	5/5	5/5	5/5	5/5	2/5	2/5	2/5	2/5	
8	5/5	5/5	5/5	5/5	5/5	5/5	2/5					
9	5/5	5/5	5/5	5/5	5/5	3/5	3/5					
10	5/5	5/5	5/5	5/5	4/5	5/5	5/5	4/5				
11	4/5	4/5	3/5	3/5								
12	3/5	4/5	4/5									
13	3/5	4/5	4/5									
14	2/5	2/5	2/5	3/5								
15	3/5	3/5										
16	3/5	3/5										
17	5/5	3/5	4/5	4/5								
18	3/5	4/5										
19												
20	4/5	3/5										
21	2/5	2/5	2/5	2/5	2/5	2/5	1/5					
22												
23	2/5											
24	5/5											
25	5/5	5/5	1/51/5									
26	2/5	1/5										
27	3/5	2/5						1/5				
28	5/5	5/5	5/5	2/5	2/5	2/5						
29	5/5	5/5	3/5	2/5	2/5	2/5						
30	3/5											
31	5/5	5/5	5/5	3/5	3/5	5/5	1/5	1/5	1/5	1/5	2/5	2/5
32	2/5	4/5	1/51/5	1/5								
33	5/5	5/5	5/5	1/5								
34	4/5	3/5	2/5	4/5								
35	5/5	3/5	4/5	1/5	1/5	1/5						
36	4/5	4/5	5/5	3/5								
37	2/5	2/5										
38												
39	1/5	3/5	1/5									
40	5/5	5/5	5/5	3/5	1/5	1/5						
41	2/5	5/5	3/5									

42	5/5	4/5	2/5	2/5	2/5							
43	1/5	1/5	1/5	2/5								
44	4/5	2/5	4/5	4/5	2/5	2/5	1/5					
45	3/5	3/5	4/5		1/5	1/5	3/5					
46	5/5	4/5	3/5	2/5	5/5	2/5						
47	5/5	5/5	2/5	1/5	3/5	4/5	3/5					
48	2/5	2/5	1/5	2/5	1/5	1/5	1/5					
49	1/5	1/5	2/5									
50	1/5		1/5									
51	2/5	3/5	3/5									
52	5/5	5/5	3/5	1/5	4/5							
53	4/5	2/5	5/5	5/5	2/5	1/5	2/5					
54	3/5	3/5	3/5									
55	4/5	4/5	2/5	1/5	3/5	2/5						
56	5/5	5/5	2/5	2/5	3/5	2/5						
57	1/5		1/5									
58	4/5	4/5	3/5	3/5	1/5							
59	4/5	2/5		1/5								
60	2/5											
61	3/5	1/5										
62		2/5	2/5									
63	1/5	1/5	1/5									
64												
65	1/5		2/5									
66	1/5		2/5									
67	1/5	1/5	1/5									
68	1/5	1/5										
69	1/5	1/5	1/5	1/5								
70	5/5	3/5	3/5	5/5	2/5							
71		2/5										
72	2/5	2/5		2/5								
73												
74		2/5	3/5									
75	1/5		2/5									
76	2/5	2/5	1/5									
77		2/5<br>3/51/5	3/5	1/5								
78												
79		2/5	3/5									
80		3/5										
81			2/5	3/5								
82	1/5	2/5	1/5	3/5	4/5							
83	2/5	2/5	2/5	5/5								
84	1/5	2/5	2/5	4/5								
85												

86	2/5	1/5	2/5	3/5								
87	4/5	5/5	3/5	3/5								
88												
89	1/5		2/5									
90	1/5		1/5									
91	1/5	1/5	1/5									
92	1/5	2/5	2/5	3/5	2/5	1/5	1/5					
93	5/5	4/5	3/5	3/5	4/5	1/5			1/5			
94	5/5	5/5										
95	5/5	5/5	5/5	5/5	4/5	2/5	4/5			1/5		
96	5/5	4/5	4/5	5/5	4/5	2/5	4/5					
97	4/5	3/5	3/5	3/5	1/5	2/5	3/5					
98	4/5	4/5	2/5	2/5								
99	1/5	1/5	1/5	3/5	1/5	1/5	2/5	2/5				
100	5/5	4/5	5/5	5/5	2/5	4/5	1/5					
101	5/5	5/5	5/5	4/5	1/5	2/5	1/5	1/5	1/5			
102	3/5	5/5	4/5									
103	5/5	5/5	3/5	2/5								
104	5/5	5/5	5/5	5/5	5/5	5/5	2/5				2/5	
105	1/5	2/5	1/5	1/5	1/5	1/5	1/5	1/5				2/5
106	5/5	5/5	4/5	5/5	4/5	4/5	4/5	4/5	2/5			
107	5/5	5/5	5/5	5/5	5/5	5/5	4/5					
108	4/5	5/5	5/5	4/5	1/5	1/5	1/5	1/5	2/5			
109	3/5	3/5	1/5									
110	5/5	5/5	5/5	4/5	5/5	2/5	5/5	1/5	2/5			2/5
111	4/5	2/5	3/5	5/5	4/5	3/5	4/5	4/5	4/5	5/5		
112	5/5	2/5	5/5	3/5	3/5	3/5	3/5	2/5	2/5	2/5		2/5
113	5/5	5/5	5/5	5/5	2/5	2/5	3/5	2/5				
114	5/5	5/5	1/5	1/5	1/5	1/5	2/5					
115	5/5	4/5	5/5	4/5	5/5	3/5	3/5					
116	5/5	5/5	5/5	5/5	5/5	3/5						
117	2/5	2/5	2/5	1/5	2/5	2/5	3/5					
118	2/5	2/5	1/5	2/5	2/5							2/5
119	2/5	2/5	2/5	1/5			2/5					
120	5/5	5/5	5/5	5/5	5/5	5/5	5/5	2/5	2/5	2/5	1/5	
121	5/5	5/5	5/5	3/5	3/5	4/5						
122	5/5	1/5	1/5	5/5	2/5	3/5						
123	5/5	4/5	5/5	5/5	5/5							
124	3/5	1/5	1/5	1/5								
125	4/5	4/5	2/5	3/5	2/5	2/5	2/5					
126	5/5	3/5	2/5									
127	2/5	2/5	2/5	3/5	1/5							
128	1/5	1/5	1/5	1/5	1/5	1/5						
129	5/5	5/5	5/5	5/5	2/5	4/5	3/5	2/5				

130	5/5	4/5	3/5	2/5	2/5	1/5	2/5	2/5				
131	5/5	4/5	2/5	2/5								
132	1/5											
133	3/5	5/5	5/5	3/5	1/5							
134												
135	5/5	2/5	2/5	1/5								
136	1/5	1/5	1/5	2/5	1/5	1/5	1/5	1/5	1/5			
137	5/5	3/5	4/5	2/5	3/5	2/5						
138	5/5	5/5	5/5	5/5	2/5	4/5				1/5		
139	3/5	2/5	2/5	1/5								
140	5/5	2/5										
141	3/5	2/5										
142	5/5	5/5	5/5	5/5	5/5	2/5	4/5	4/5	5/5	5/5		
143	5/5	5/5	5/5	5/5	5/5	3/5						
144	5/5	5/5	1/5	5/5	3/5	2/5						
145	5/5	5/5	5/5	2/5								
146	5/5	5/5	5/5	5/5	5/5	5/5	1/5	1/5				
147	2/5	5/5	1/5	4/5								
148	5/5	5/5	3/5	5/5	3/5	2/5	3/5	4/5				
149	2/5	2/5										
150	3/5	1/5	1/5	1/5	2/5	2/5	2/5					
151	4/5	4/5	2/5	2/5	2/5	2/5	2/5					
152	5/5	3/5	2/5	3/5								
153	3/5	5/5	5/5	2/5	5/5	1/5						
154	5/5	5/5	5/5	5/5	5/5	3/5						
155	5/5	3/5	2/5									
156	3/5	1/5										
157	5/5	5/5	5/5	5/5	1/5	4/5	3/5					
158	5/5	5/5	3/5	5/5	5/5	5/5						
159	5/5	2/5	2/5	2/5	2/5	2/5				2/5		
160	5/5	5/5	5/5	3/5								
161	4/5	3/5	4/5	2/5	1/5	1/5	2/5	2/5	2/5	3/5		
162	5/5	1/5	5/5	2/5	1/5	1/5						
163	5/5	5/5	2/5	5/5	3/5	4/5						
164	4/5	4/5	2/5									
165	5/5	3/5	5/5	4/5	3/5	2/5	1/5					
166	5/5	5/5	5/5	5/5								
167	5/5	5/5	5/5	5/5	5/5	4/5	2/5				2/5	
168	2/5	2/5										
169	3/5	2/5	3/5	4/5	3/5	1/5						
170	4/5	3/5	4/5	3/5	2/5	1/5	2/5					
171	5/5	5/5	5/5	5/5	5/5	4/5						
172	5/5	1/5	1/5			1/5						
173	5/5	5/5	4/5	2/5								

174												
175												
176	5/5											
177	5/5	5/5	5/5	5/5	5/5	1/5						
178	3/5	3/5	2/5	3/5								
179												
180	5/5	1/5	5/5	2/5								
**Norstar**	5/5	5/5	5/5	5/5	5/5	5/5	5/5	5/5	5/5	5/5	3/5	2/5
**Zagros**	4/5	2/5										
