# Supplementary material for: Identification of Novel QTLs Associated with Frost Tolerance in Winter Wheat (Triticum aestivum L.)
Source: Plants (Basel). 2023 Apr 13;12(8):1641. doi: 10.3390/plants12081641 (PMC10146367; doi:10.3390/plants12081641)
Supplement: Supplementary file 1 [file plants-12-01641-s001.zip › plants-2247413-supplementary S2.pdf]

Table S2. 425- SSR primers for QTL analysis among RIL accessions.

Primer No.	Marker Name	Forward (5′–3′)	Backward (5′–3′)	Location
1	BARC3	TTCCCTGTGTCTTTCTAATTTTT	GCGAACTCCCGAACATTTTTAT	6A
2	BARC2	GCGATCCACACTTTGCCTCTTTT	GCGATGTCGGTTTTCAGCCTTTT	4B, 7B
3	BARC45	CCCAGATGCAATGAAACCACA	GCGTAGAACTGAAGCGTAAAATT	3A, 2B
4	BARC58	GCGACTTTGTGTATATGTTTTA	ATGGGGCAATTAGTGTTTTCTTCG	5BL-7DS
5	BARC64	GCGGAGTCTGCAATTAGTATAG	GCATCCACCTCCGCAGTCAGT	7A
6	BARC66	CGCGATCGATCTCCCGGTTTGC	GGGAAGAGGACCAAGGCCACTA	1DL,2B
7	BARC96	AAGCCTTGTTGTTCCGTATTATT	GCGGTTTATATTTGTGGTTGAGC	6DL6-5B
8	BARC98	CCGTCCTATTCGCAAACCAGAT	GCGGATATGTTCTCTAACTCAAGC	4D,2B
9	BARC105	CAGGAAGAAAAGGAAAGCATG	GCGGTGTGGCAATAATTACTTTTT	7A-4D
10	BARC117	TCATGCGTGCTAAGTGCTAA	GAGGGCAGGAAAAAGTGACT	5A
11	BARC118	AGTTGCCGCTTCTTTTCATTTTT	AGAGGTCCATTTTTCGTCCTTTGA	4D-6A
12	BARC119	CACCCGATGATGAAAAT	GATGGCACAAGAAATGAT	3D, 1A, 1B,
13	BARC122	CCCGTGTATATCCAGGAGTG	CAGCCCTTGTGATGTGATG	5AL12,-2A
14	BARC124	TGCACCCCTTCCAAATCT	TGCGAGTCGTGTGGTTGT	5A, 2B
15	BARC126	CCATTGAAACCGGATTTGAGTC	CGTTCCATCCGAAATCAGCAC	7D4-7B
16	BARC127	TGCATGCACTGTCCTTTGTATT	AAGATGCGGGCTGTTTTCTA	7A-, 6B
17	BARC127	TGCATGCACTGTCCTTTGTATT	AAGATGCGGGCTGTTTTCTA	-
18	BARC13	CGGCTAGTAGTTGGAGTGTTGG	ACCGCCTCTAGTTATTGCTCTC	5DS2-0.78
19	BARC133	AGCGCTCGAAAAGTCAG	GGCAGGTCCAACTCCAG	5DL-3BS
20	BARC134	CCGTGCTGCAAATGAACAC	AGTTGCCGGTTCCCATTGTCA	6BS
21	BARC138	CTCGATTCGCCGTCAG	GTGGGGGAAGAAGAAACC	2A, 4A
22	BARC138	CTCGATTCGCCGTCAG	GTGGGGGAAGAAGAAACC	-
23	BARC140	CGCCAACACCTACCATT	TTCTCCGCACTCACAAAC	5BL,2B, 5D
24	BARC142	CCGGTGAGAGGACTAAAA	GGCCTGTCAATTATGAGC	5BL,2D, 5A,
25	BARC145	GCAGCCTCGAATCACA	GGGGTGTTGAAGATGA	2D, 3B, 1A
26	BARC146	AAGGCGATGCTGCAGCTAAT	GGCAATATGGAAACTGGAGAGAA	6D,-6A, 6B
27	BARC147	GCGCCATTTATTCATGTTCCTC	CCGCTTCACATGCAATCCGTTGAT	3B
28	BARC148	GCGCAACCACAATGTATGCT	GGGGTGTTTTCCTATTTCTT	C-1A-1D,
29	BARC149	ATTCACTTGCCCCTTTTAAACTC	GAGCCGTAGGAAGGACATCTAGT	1D
30	BARC154	GTAATTCCGGTTCCACTTGACA	GGATGGGCAGCTTCAAGGTATGT	7D,7A
31	BARC203	GCGAACATGTATCCAAGTCACT	GCGCCACATTCAAACATAAGGTC	3B
32	BARC206	GCTTTGCCAGGTGAGCACTCT	TGGCCGGGTATTTGAGTTGGAGTT	4A,3B, 6A
33	BARC232	CGCATCCAACCATCCCCACCCA	CGCAGTAGATCCACCACCCCGCC	5A, 5B, 5D
34	BARC1090	CGCTCCAATGATGACACC	CGGAAGGAAGAAGAAGAAGA	1D
35	BARC1099	CCCACGCACGCACTAAG	TCATGTGGCCGTTGTGAA	3A, 6B
36	CFA2040	TCAAATGATTTCAGGTAACCAC	TTCCTGATCCCACCAAACAT	7D,7A, 7B,
37	CFA2049	TAATTTGATTGGGTCGGAGC	CGTGTCGATGGTCTCCTTG	7A

38	CFA2056	TGGAAGTTACCCTGTCCGTC	GAAGAGAGGAGGAGTGGCCT	1A, 7A
39	CFA2058	CCCATTGCCATCTCAGTCTT	ATAGTAGGCCCAAAGCGATG	2A
40	CFA2091	CCTTCTTAATGACCGGCTCG	AAACGAAGACGTGGACATGG	4B
41	CFA2106	GCTGCTAAGTGCTCATGGTG	TGAAACAGGGGAATCAGAGG	7B
42	CFA2114	ATTGGAAGGCCACGATACAC	CCCGTCGGGTTTTATCTAGC	6A
43	CFA2129	GTTGCACGACCTACAAAGCA	ATCGCTCACTCACTATCGGG	1A, 1B, 1D
44	CFA2135	TGCCTAAATCTAAATGCCCG	GGATAATGTGCATGTTCACCG	1A
45	CFA2153	TTGTGCATGATGGCTTCAAT	CCAATCCTAATGATCCGCTG	1A
46	CFA2155	TTTGTTACAACCCAGGGGG	TTGTGTGGCGAAAGAAACAG	5A
47	CFA2190	CAGTCTGCAATCCACTTTGC	AAAAGGAAACTAAAGCGATGGA	5A
48	CFA2219	TCTGCCGAGTCACTTCATTG	GACAAGGCCAGTCCAAAAGA	1A
49	CFA2240	TGCAGCATGCATTTTAGCTT	TGCCGCACTTATTTGTTCAC	7A
50	CFA2241	TTGGCCATCAGGCTCTAGTT	GTGATGCTGTTCTCAAGCCA	1B
51	CFA2250	AGCCATAGATGGCCCTACCT	CACTCAATGGCAGGTCCTTT	5A
52	CFA2278	GCCTCTGCAAGTCTTTACCG	AAGTCGGCCATCTTCTTCCT	2B
53	CFA2292	GGACCGTTTATCCGTAAGCA	GCCTATGCTGCTGATCCATT	1B
54	CFD1	ACCAAAGAACTTGCCTGGTG	AAGCCTGACCTAGCCCAAAT	6A, 6B,6D
55	CFD4	TGCTCCGTCTCCGAGTAGAT	GGGAAGGAGAGATGGGAAAC	3D, 3BL
56	CFD5	TGCCCTGTCCACAGTGAAG	TTGCCAGTTCCAAGGAGAAT	6D, 5B
57	CFD9	TTGCACGCACCTAAACTCTG	CAAGTGTGAGCGTCGG	3D
58	CFD10	CGTTCTATGACGTGTCATGCT	TCCATTTTCAAAAACACCCTG	5D
59	CFD13	CCACTAACCAAGCTGCCATT	TTTTTGGCATTGATCTGCTG	6D-6B
60	CFD16	GGATCCAAGGGAATCCAAAT	TCCTTCGGTTCCCATATCAC	4A-2D
61	CFD35	GGGATGACACATAACGGACA	ATCAGCGGCGCTATAGTACG	3D
62	CFD036	GCAAAGTGTAGCCGAGGAAG	TTAGAGTTTTGCAGCGCCTT	2D-2A
63	CFD042	AGGTTCTAGGGGGCATGTCT	GCTCTCAATGACTGCACTGG	6D
64	CFD043	AACAAAAGTCGGTGCAGTCC	CCAAAAACATGGTTAAAGGGG	2D
65	CFD045	TCTCTCCAGTTGCTCCTCGT	ATGTGGAACCGGTCTACTCG	6D
66	CFD048	ATGGTTGATGGTGGGTGTTT	ATGTATCGATGAAGGGCCAA	1B, 1D
67	CFD049	TGAGTTCTTCTGGTGAGGCA	GAATCGGTTCACAAGGGAAA	6D
68	CFD051	GGAGGCTTCTCTATGGGAGG	TGCATCTTATCCTGTGCAGC	2D
69	CFD054	TCGTTCCAAAATGCATGAAA	AAGGGCCAGAAATCTGTGTG	4BL54D
70	CFD059	TCACCTGGAAAATGGTCACA	AAGAAGGCTAGGGTTCAGGC	1A, 1B, 1D
71	CFD060	TGACCGGCATTCAGTATCAA	TGGTCACTTTGATGAGCAGG	6D
72	CFD061	ATTCAAATGCAACGCAAACA	GTTAGCCAAGGACCCCTTTC	1D
73	CFD066	AGGTCTTGGTGGTTTTGGTG	TTTTCACATGCCCACAGTTG	7D
74	CFD068	TTTGCAGCATCACACGTTTT	AAAATTGTATCCCCCGTGGT	7DL, 7A
75	CFD073	GATAGATCAATGTGGGCCGT	AACTGTTCTGCCATCTGAGC	2D, 2B
76	CFD074	TCAAAACCACACCAGGCATA	AAGTGGTGGGGAGTGTGTGT	5D, 7B
77	CFD079	TCTGGTTCTTGGGAGGAAGA	CATCCAACAATTTGCCCAT	3D,3B
78	CFD080	ATAGGGGTTTTGAATCACTCC	TTGGATTTGCAGAGCCTTCT	6A, 6D

79	CFD082	GCTGATGCTGCTGTAAGTGC	TGAAGAATACAATGGCAGCAA	6A
80	CFD088	TAGGCATAGTTTTGGGCCTG	GGTAGAAGGAAGCTTCGGGA	4A
81	CFD106	ACGGGTGGTTTTGCTCAGT	ACTCCACCAGCGGAGAAATA	4D
82	CFD135	GGATCTCGGGGATGTCCT	TAAGCACCTTCTTCATGGGG	6D
83	CFD141	CGTAAAGATCCGAGAGGGTG	TCCGAGGTGCTACCTACCAG	3D
84	CFD161	GTAAGGCATCTTCGCGTCTC	CCATGATAGATTTGGACGGG	2D
85	CFD170	CTGTCGGACGACGACGA	CATCCTCTTGACGCCGCCGC	1B
86	CFD175	TGTCGGGGACACTCTCTCTT	ACCAATGGGATGCTTCTTTG	2D, 7D
87	CFD189	GCTAAAGCCACATAGGACGG	GCACAAGATTTTGCAAGGCT	5D
88	CFD211	AGAAGACTGCACGCAAGGAT	TGCACTAAAGCATCTTCGTGTT	3D
89	CFD239	CTCTCGTTCTCTCCAGGCTC	GAGAGGAGAGCTTGCCATTG	2D
90	CFD239	CTCTCGTTCTCTCCAGGCTC	GAGAGGAGAGCTTGCCATTG	-
91	CFD267	GTGCGTCGTGTAGCAGCTC	CTCTCTGTCGTCCAGGTCGT	2A, 2D
92	GDM033	GGCTCAATTCAACCGTTCTT	TACGTTCTGGTGGCTGCTC	1A, 1B, 1D
93	GDM046	TGTGTTGGCCTTGTGGTG	CTACCCAATGCATCCCCTTA	7D
94	GDM060	AGAAGGACCGTCGCGTC	ATGTTGGCCCTGAAGAAGAA	1D
95	GDM067	AAGCAAGGCACGTAAAGAGC	CTCGAAGCGAACACAAAACA	7D
96	GDM072	TGGTTTTCTCGAGCATTCAA	TGCAACGATGAAGACCAGAA	3D
97	GDM098	CCATCCATGAAATGGCG	GCCCTTCACTAGCCTTCATG	6D
98	GDM109	GGTCCGCCTGACAGACC	AAAGCTGCTCATCGTGGTG	5A
99	GDM152	ATAACATGCACACAAATTTT	GCCAGTGCCAAGCTTGC	7A
100	GWM011	GGATAGTCAGACAATTCTTGTG	GTGAATTGTGTCTTGTATGCTTCC	1B
101	GWM033	GGAGTCACACTTGTTTGTGCA	CACTGCACACCTAACTACCTGC	-
102	GWM044	GTTGAGCTTTTCAGTTCGGC	ACTGGCATCCACTGAGCTG	4A,7D
103	GWM046	GCACGTGAATGGATTGGAC	TGACCCAATAGTGGTGGTCA	7B
104	GWM052	CTATGAGGCGGAGGTTGAAG	TGCGGTGCTCTTCCATT	3D
105	GWM063	TCGACCTGATCGCCCCTA	CGCCCTGGGTGATGAATAGT	7A
106	GWM067	ACCACACAAACAAGGTAAGCG	CAACCCTCTTAATTTTGTTGGG	5B
107	GWM070	AGTGGCTGGGAGAGTGTCAT	GCCCATTACCGAGGACAC	6B
108	GWM088	CACTACAACTATGCGCTCGC	TCCATTGGCTTCTCTCTCAA	6B
109	GWM095	CACTACAACTATGCGCTCGC	TCCATTGGCTTCTCTCTCAA	2A
110	GWM099	AAGATGGACGTATGCATCACA	GCCATATTTGATGACGCATA	-
111	GWM102	TCTCCCATCCAACGCCTC	TGTTGGTGGCTTGACTATTG	-
112	GWM121	TCCTCTACAAACAAACACAC	CTCGCAACTAGAGGTGTATG	7D
113	GWM122	GGGTGGGAGAAAGGAGATG	AAACCATCCTCCATCCTGG	2B
114	GWM124	GCCATGGCTATCACCCAG	ACTGTTCGGTGCAATTTGAG	1B
115	GWM126	CACACGCTCCACCATGAC	GTTGAGTTGATGCGGGAGG	5A
116	GWM129	TCAGTGGGCAAGCTACACAG	AAAACTTAGTAGCCGCGT	1B
117	GWM132	TACCAAATCGAAACACATGAGG	CATATCAAGGTCTCCTTCCCC	2B,6A
118	GWM133	ATCTAAACAAGACGGCGGTG	ATCTGTGACAACCGGTGAGA	1B,3A
119	GWM136	GACAGCACCTTGCCCTTTG	CATCGGCAACATGCTCATC	1A

120	GWM140	ATGGAGATATTTGGCCTACAAC	CTTGACTTCAAGGCGTGACA	1B
121	GWM146	CCAAAAAAACTGCCTGCATG	CTCTGGCATTGCTCCTTGG	7B
122	GWM156	CCAACCGTGCTATTAGTCATTC	CAATGCAGGCCCTCCTAAC	4B,5B
123	GWM160	TTCAATTCAGTCTTGGCTTGG	CTGCAGGAAAAAAAGTACACCC	4A
124	GWM161	GATCGAGTGATGGCAGATGG	TGTGAATTACTTGGACGTGG	3D
125	GWM181	TCATTGGTAATGAGGAGAGA	GAACCATTCATGTGCATGTC	3B
126	GWM183	GTCTTCCCATCTCGCAAGAG	CTCGACTCCCATGTGGATG	3D
127	GWM186	GCAGAGCCTGGTTCAAAAAG	CGCCTCTAGCGAGAGCTATG	5A
128	GWM192	GGTTTTCTTTCAGATTGCGC	CGTTGTCTAATCTTGCCTTGC	4A,4B
129	GWM194	GATCTGCTCTACTCTCCTCC	CGACGCAGAACTTAAACAAG	4D
130	GWM205	CGACCCGGTTCACTTCAG	AGTCGCCGTTGTATAGTGCC	5A,5D
131	GWM210	TGCATCAAGAATAGTGTGGAAG	TGAGAGGAAGGCTCACACCT	2A,2B
132	GWM213	TGCCTGGCTCGTTCTATCTC	CTAGCTTAGCACTGTCGCCC	4D,4B
133	GWM219	GATGAGCGACACCTAGCCTC	GGGGTCCGAGTCCACAAC	6B
134	GWM221	ATGAGCGACACCTGCCTCC	GGGTCCGAGTCCACAAA	7D
135	GWM232	ATCTCAACGGCAAGCCG	CTGATGCAAGCAATCCAC	1D
136	GWM233	TCAAAACATAAATGTTCATTGGA	TCAACCGTGTGTAATTTTGTCC	7A
137	GWM234	GAGTCCTGATGTGAAGCTGTTG	CTCATTGGGGTGTGTACGTG	5A,5B
138	GWM247	GCAATCTTTTTTCTGACCACG	ATGTGCATGTCGGACGC	3A,3B
139	GWM257	AGAGTGCATGGTGGGACG	CCAAGACGATGCTGAAGTCA	2B
140	GWM260	GCCCCCTTGCACAATC	CGCAGCTACAGGAGGCC	7A
141	GWM271	CAAGATCGTGGAGCCAGC	AGCTGCTAGCTTTTGGGAC	2B,5B,5D
142	GWM276	ATTTGCCTGAAGAAAATATT	AATTTCACTGCATACACAA	7A,7B
143	GWM282	TTGGCCGTGTAAGGCAG	TCTCATTCACACACAACACTAGC	7A
144	GWM292	TCACCGTGGTCACCGAC	CCACCGAGCCGATAATGTAC	5D
145	GWM294	GGATTGGAGTTAAGAGAGAACCG	GCAGAGTGATCAATGCCAGA	2A
146	GWM295	GTGAAGCAGACCCACAACAC	GACGGCTGCGACGTAGAG	7D
147	GWM301	GAGGAGTAAGACACATGCCC	GTGGCTGGAGATTCAGGTTC	2D
148	GWM312	ATCGCATGATGCACGTAGAG	ACATGCATGCCTACCTAATGG	2A
149	GWM314	AGGAGCTCCTCTGTGCCAC	TTCGGGACTCTCTTCCCTG	3D
150	GWM334	AATTTCAAAAAGGAGAGAGA	AACATGTGTTTTTAGCTATC	6A
151	GWM335	CGTACTCCACTCCACACGG	CGGTCCAAGTGCTACCTTTC	5B
152	GWM340	GCAATCTTTTTTCTGACCACG	ACGAGGCAAGAACACACATG	3B
153	GWM350	ACCTCATCCACATGTTCTACG	GCATGGATAGGACGCCC	4A
154	GWM368	CCATTTCACCTAATGCCTGC	AATAAAACCATGAGCTCACTTGC	4B
155	GWM369	CTGCAGGCCATGATGATG	ACCGTGGGTGTTGTGAGC	3A,4B
156	GWM372	AATAGAGCCCTGGGACTGGG	GAAGGACGACATTCCACCTG	2A
157	GWM374	ATAGTGTGTTGCATGCTGTGTG	TCTAATTAGCGTTGGCTGCC	1B,2B
158	GWM382	GTCAGATAACGCCGTCCAAT	CTACGTGCACCACCATTTTG	2A,2B,2D
159	GWM391	ATAGCGAAGTCTCCCTACTCCA	ATGTGCATGTCGGACGC	3A
160	GWM400	GTGCTGCCACCACTTGC	TGTAGGCACTGCTTGGGAG	7B

161	GWM403	CGACATTGGCTTCGGTG	ATAAAACAGTGCGGTCCAGG	1B,2B
162	GWM410	GCTTGAGACCGGCACAGT	CGAGACCTTGAGGGTCTAGA	5B,5A
163	GWM413	TGCTTGTCTAGATTGCTTGGG	GATCGTCTCGTCCTTGGCA	1B
164	GWM415	GATCTCCCATGTCCGCC	CGACAGTCGTCACTTGCCTA	5A
165	GWM427	AAACTTAGAACTGTAATTTCAGA	AGTGTGTTCATTTGACAGTT	6A
166	GWM441	AACTTAGAACTGTAATTTCAGAG	GTGTGTTCATTTGACAGTTT	2B
167	GWM428	CGAGGCAGCGAGGATTT	TTCTCCACTAGCCCCGC	7D
168	GWM459	ATGGAGTGGTCAACATTTGAA	AGCTTCTCTGACCAACTTCTCG	6A
169	GWM469	CAACTCAGTGCTCACACAACG	CGATAACCACTCATCCACACC	-
170	GWM480	TGCTGCTACTTGTACAGAGGAC	CCGAATTGTCCGCCATAG	3A
171	GWM493	TTCCCATAACTAAAACCGCG	GCAACATCATTTCTGGACTTTG	3B
172	GWM494	ATTGAACAGGAAGACATCAGGG	TTCCTGGAGCTGTCTGGC	1B,3A,4A
173	GWM497	GTAGTGAAGACAAGGGCATT	CCGAAAGTTGGGTGATATA	1A
174	GWM501	GGCTATCTCTGGCGCTAAAA	TCCACAAACAAGTAGCGCC	2B
175	GWM508	GTTATAGTAGCATATAATGGCC	GTGCTGCCATGATATTT	6B
176	GWM518	AATCACAACAAGGCGTGACA	CAGGGTGGTGCATGCAT	6B
177	GWM533	AAGGCGAATCAAACGGAATA	GTTGCTTTAGGGGAAAAGC	3B,3D
178	GWM538	GCATTTCGGGTGAACCC	GTTGCATGTATACGTTAAGC	4B
179	GWM565	GCGTCAGATATGCCTACCTAGG	AGTGAGTTAGCCCTGAGCC	4A,5D
180	GWM569	GGAAACTTATTGATTGAAAT	TCAATTTTGACAGAAGAAT	7B
181	GWM570	TCGCCTTTTACAGTCGGC	ATGGGTAGCTGAGAGCCAA	6A
182	GWM583	TTCACACCCAACCAATAGCA	TCTAGGCAGACACATGCCT	5D
183	GWM608	ACATTGTGTGTGCGGCC	GATCCCTCTCCGCTAGAAG	1B,1D,2D
184	GWM609	GCGACATGACCATTTTGTTG	GATATTAAATCTCTCTATGT	4D
185	GWM611	CATGGAAACACCTACCGAAA	CGTGCAAATCATGTGGTAG	7B
186	GWM613	CCGACCCGACCTACTTCTCT	TTGCCGTCGTAGACTGG	4A
187	GWM613	CCGACCCGACCTACTTCTCT	TTGCCGTCGTAGACTGG	6B
188	GWM630	GTGCCTGTGCCATCGTC	CGAAAGTAACAGCGCAGTG	2B
189	GWM635	TTCCTCACTGTAAGGGCGTT	CAGCCTTAGCCTTGGCG	7A
190	GWM636	CGGTAGTTTTTAGCAAAGAG	CCTTACAGTTCTTGGCAGAA	7A,7B
191	GWM637	AAAGAGGTCTGCCGCTAACA	TATACGGTTTTGTGAGGGG	4A
192	GWM639	CTCTCTCCATTCGGTTTTCC	CATGCCCCCCTTTTCTG	5A
193	GWM642	ACGGCGAGAAGGTGCTC	CATGAAAGGCAAGTTCGTC	5A,5B,5D
194	GWM644	GTGGGTCAAGGCCAAGG	AGGAGTAGCGTGAGGGGC	1D
195	GWM645	TGACCGGAAAAGGGCAGA	GCCCCTGCAGGAGTTTAAG	3D
196	WMC010	GATCCGTTCTGAGGTGAGTT	GGCAGCACCCTCTATTGTCT	7B
197	WMC048	GAGGGTTCTGAAATGTTTTGCC	ACGTGCTAGGGAGGTATCT	4B,4D
198	WMC163	TTACACCCATCAGGGTGGTCTT	GTCTATCCATACGACAA	6A
199	WMC285	TGTGGTTGTATTTGCGGTATGG	TTGTGGTGCTGAGTTAGCTT	4D
200	WMC326	GGAGCATCGCAGGACAGA	GGACGAGGACGCCTGAAT	3B,5B
201	WMC329	ACAAAGGTGCATTCGTAGA	AACACGCATCAGTTTCAGT	1A,1B

202	WMC356	GCCGTTGCCCAATGTAGAAG	CCAGAGAAACTCGCCGTGTC	2B
203	WMC376	TCTCAACCACCGACTTGTAA	ACATGTAATTGGGGACACTG	3B
204	WMC506	CACTTCCTCAACATGCCAGA	CTTTCAATGTGGAAGGCGAC	7D
205	WMC511	CGCACTCGCATGATTTTCCT	ATGCCCGGAAACGAGACTGT	4B
206	WMC532	GATACATCAAGATCGTGCCAAA	GGGAGAAATCATTAACGAAGGG	3A
207	WMC557	GGTGCTTGTTCATACGGGCT	AGGTCCTCGATCCGCTCAT	7B
208	WMC559	ACACCACGAATGATGTGCCA	ACGACGCCATGTATGCAGAA	3A
209	WMC594	CCCCTCACTGCCG	ATATCCATATAGTACTCGCAC	3A
210	WMC622	CAGGAAGAAGAGCTCCGAGAAA	CTTGCTAACCCGCGCC	4D
211	WMC687	AGGACGCCTGAATCCGAG	GGGAGCGTAGGAGGACTAACA	3B
212	WMC710	GTAAGAAGGCAGCACGTATGAA	TAAGCATTCCCAATCACTCTCA	4B
213	WMC722	GCTTTTCGATGGGATGGTGC	TTTGTCCACTGCCTTCTGCC	4A
214	WMC764	CCTCGAACCTGAAGCTCTGA	TTCGCAAGGACTCCGTAACA	2B
215	WMC765	GGGATCAGACTGGGACTGGAG	GGGTTGGCTTGGCAGAGAA	5D
216	WMC776	CCATGACGTGACAACGCAG	ATTGCAGGCGCGTTGGTA	1B
217	WMC783	AGGTTGGAGATGCAGGTGGG	TCTTCCTTCTCCTGCCGCTA	5D
218	GWM 261	GATGTGCATGTGAATCTCAAAAGTA	AAAGAGGGTCACAGAATAACCTAAA	D2
219	GWM 271	ACTACTTAGGCCTCCCGCC	TGACCCACTTGCAATTCATC	D5-B2-B5
220	GWM 272	GCAAGAAGCAACAGCAGTAAC	' CAGATGCTCTTCTCTGCTGG	D5
221	GWM 274	AGGAAACAGAAATATCGCGG	AGGACTGTGGGGAATGAATG	B3-B5
222	GWM 276	TCACGTGGAAGACGCTCC	CTACGTGCACCACCATTTTG	A7
223	GWM 282	GGTTGCTGTACAAGTGTTCACG	CGGGTGCTGTGTGTAATGAC	A7
224	GWM 292	TTTCTTCTGTCGTTCTCTTCCC	' TTTTTACGCGTCAACGACG	D5
225	GWM 296	AGCCAGCAAGTCACCAAAAC	AGTGCTGGAAAGAGTAGTGAAGC	-
226	GWM 299	' GCCCGGTCATGTAAAACG	TTTCAGTTTGCGTTAAGCTTTG	B3-B2
227	GWM 302	' CGTACTCCACTCCACACGG	CGGTCCAAGTGCTACCTTTC	B7
228	GWM 304	CCTCTTCCTCCCTCACTTAGC	' TGCTAACTGGCCTTTGCC	A2-A5
229	GWM 311	CAAGGAAATAGGCGGTAACT	ATTTGAGTCTGAAGTTTGCA '	A2
230	GWM 319	GGCTTCCAGAAAACAACAGG	ATCGGTGCGTACCATCCTAC	B2
231	GWM 325	TATGGTCAAAGTTGGACCTCG	AGGCTGCAGCTCTTCTTCAG	D6
232	GWM 332	AAACAGCGGATTTCATCGAG	TCCGCTGTTGTTCTGATCTC	A7
233	GWM 333	CCATTTCACCTAATGCCTGC	AATAAAACCATGAGCTCACTTGC	B7
234	GWM 335	CTGCAGGCCATGATGATG	ACCGTGGGTGTTGTGAGC	B5
235	GWM 337	GACCAAGATATTCAAACTGGCC	AGCTCAGCTTGCTTGGTACC	B1-D1
236	GWM 344	ATAGTGTGTTGCATGCTGTGTG	TCTAATTAGCGTTGGCTGCC	B7
237	GWM 349	GTCAGATAACGCCGTCCAAT	CTACGTGCACCACCATTTTG	D2
238	GWM 357	CTACAATTCGAAGGAGAGGGG	CACCGCGTCAACTACTTAAGC	A1
239	GWM 358	ATCATGTCGATCTCCTTGACG	TGCCATGCACATTAGCAGAT	D5
240	GWM 368	TGTCATGGATTATTTGGTCGG	CTGCACTCTCGGTATACCAGC	B4
241	GWM 369	GTGCTGCCACCACTTGC	TGTAGGCACTGCTTGGGAG	A3
242	GWM 371	TCGATTTATTTGGGCCACTG	GTATAATTCGTTCACAGCACGC	B5

243	GWM 374	GCTTGAGACCGGCACAGT	CGAGACCTTGAGGGTCTAGA	B2-B1
244	GWM 191	AGACTGTTGTTTGCGGGC	TAGCACGACAGTTGTATGCATG	B2-B6
245	GWM 192	GGTTTTCTTTCAGATTGCGC	CGTTGTCTAATCTTGCCTTGC	D5
246	GWM 194	GATCTGCTCTACTCTCCTCC	CGACGCAGAACTTAAACAAG	D4
247	GWM212	AAGCAACATTTGCTGCAATG	TGCAGTTAACTTGTTGAAAGGA	D5
248	GWM 13	TGCCTGGCTCGTTCTATCTC	CTAGCTTAGCACTGTCGCCC	B5
249	GWM219	GATGAGCGACACCTAGCCTC 3'	GGGGTCCGAGTCCACAAC	B6
250	WMS 2	CTGCAAGCCTGTGATCAACT	CATTCTCAAATGATCGAACA	A3-D3
251	WMS 5	GCCAGCTACCTCGATACAACTC	AGAAAGGGCCAGGCTAGTAGT	A3
252	WMS 6	CGTATCACCTCCTAGCTAAACTAG	AGCCTTATCATGACCCTACCTT	A5-B4-
253	WMS 10	CGCACCATCTGTATCATTCTG	TGGTCGTACCAAAGTATACGG	A2-B2
254	WMS 16	GCTTGGACTAGCTAGAGTATCATA	CAATCTTCAATTCTGTCGCACGG	B2-D5
255	WMS 24	CACACAAGGCACCATTGC	CAATGGACATAGTTGTGTGCG	D2-B1
256	WMS 30	ATCTTAGCATAGAAGGGAGTGGG	TTCTGCACCCTGGGTGAT	A2,A4
257	WMS 32	TATGCCGAATTTGTGGACAA	TGCTTGGTCTTGAGCATCAC	A3
258	WMS 33	GGAGTCACACTTGTTTGTGCA	CACTGCACACCTAACTACCTGC	A1-B1
259	WMS 37	ACTTCATTGTTGATCTTGCATG	CGACGAATTCCCAGCTAAAC	D7- D2
260	WMS 43	CACCGACGGTTTCCCTAGAGT	GGTGAGTGCAAATGTCATGTG	B7-A4
261	WMS 44	GTTGAGCTTTTCAGTTCGGC	ACTGGCATCCACTGAGCTG	D7-A4
262	WMS 46	GCACGTGAATGGATTGGAC	TGACCCAATAGTGGTGGTCA	B7
263	WMS 47	TTGCTACCATGCATGACCAT'	TTCACCTCGATTGAGGTCCT	B2-A2
264	WMS 52	CTATGAGGCGGAGGTTGAAG	TGCGGTGCTCTTCCATTT	D3
265	WMS 55	GCATCTGGTACACTAGCTGCC	TCATGGATGCATCACATCCT	B2-D6
266	WMS 58	TCTGATCCCGTGAGTGTAACA	GAAAAAAATTGCATATGAGCCC	B6
267	WMS 63	TCGACCTGATCGCCCCTA	CGCCCTGGGTGATGAATAGT	A7
268	WMS 67	ACCACACAAACAAGGTAAGCG	CAACCCTCTTAATTTTGTTGGG	A3-B5
269	WMS 72	TGGTCCCTCTCCCTTTCTCT	ACAGAATTGAAGATTGTCGGTC	B3
270	WMS 77	ACAAAGGTAAGCAGCACCTG	ACCCTCTTGCCCGTGTTG	B3
271	WMS 88	CACTACAACTATGCGCTCGC	TCCATTGGCTTCTCTCTCAA	B6
272	WMS 95	GATCAAACACACACCCCTCC	AATGCAAAGTGAAAAACCCG	A2
273	WMS 99	AAGATGGACGTATGCATCACA	GCCATATTTGATGACGCATA	A1
274	WMS 102	TCTCCCATCCAACGCCTC	'TGT TGG TGG CTT GAC TAT TG	D2
275	WMS 106	CTGTTCTTGCGTGGCATTAA	AATAAGGACACAATTGGGATGG	D1
276	WMS 107	ATTAATACCTGAGGGAGGTGC	GGTCTCAGGAGCAAGAACAC	B4-B3-B6
277	WMS 108	CGACAATGGGGTCTTAGCAT	TGCACACTTAAATTACATCCGC	B3-B2
278	WMS 111	TCTGTAGGCTCTCTCCGACTG	ACCTGATCAGATCCCACTCG	D7-A4-B7-
279	WMS 113	ATTCGAGGTTAGGAGGAAGAGG	GAGGGTCGGCCTATAAGACC	B4
280	WMS 114	ACAAACAGAAAATCAAAACCCG	ATCCATCGCCATTGGAGTG 3'	B3-D3
281	WMS 118	GATGTTGCCACTTGAGCATG	GATTAGTCAAATGGAACACCCC	A4-B5
282	WMS 120	GATCCACCTTCCTCTCTCTC	GATTATACTGGTGCCGAAAC	B2
283	WMS 121	TCCTCTACAAACAAACACAC	CTCGCAACTAGAGGTGTATG	D5-D7

284	WMS 122	-	-	-
285	WMS 124	GCCATGGCTATCACCCAG	ACTGTTCGGTGCAATTTGAG	B1
286	WMS 126	CACACGCTCCACCATGAC	GTTGAGTTGATGCGGGAGG	A5
287	WMS 129	TCAGTGGGCAAGCTACACAG	AAAACTTAGTAGCCGCGT	B2-A5
288	WMS 140	ATGGAGATATTTGGCCTACAAC	CTTGACTTCAAGGCGTGACA	B1
289	WMS 148	GTGAGGCAGCAAGAGAGAAA	CAAAGCTTGACTCAGACCAAA	B2
290	WMS 149	CATTGTTTTCTGCCTCTAGCC	CTAGCATCGAACCTGAACAAG	B4
291	WMS 155	CAATCATTTCCCCCTCCC	AATCATTGGAAATCCATATGCC	A3
292	WMS 174	GGGTTCCTATCTGGTAAATCCC	GACACACATGTTCCTGCCAC	D5
293	WMS 181	TCATTGGTAATGAGGAGAGA	GAACCATTCATGTGCATGTC	B3
294	WMS 186	GCAGAGCCTGGTTCAAAAAG	CGCCTCTAGCGAGAGCTATG	A5
295	WMS 189	AGGAGCAGCGGAACGAAC	AGAAATACGGAAACCCACCC	B2
296	WMS 190	GTGCTTGCTGAGCTATGAGTC	GTGCCACGTGGTACCTTTG	D5
297	WMS 200	TCAACGGAACAGATGAGCG	GACCTGATGAGAGCAAGCAC	-
298	WMS 210	TGCATCAAGAATAGTGTGGAAG	TGAGAGGAAGGCTCACACCT	-
299	WMS 388	GCGATGCTTTTGCCTTGTTTCAG	GCGGCCCCTTTGACTCTTCATAG	-
300	BARC 1	TTCCCTGTGTCTTTCTAATTTTTTT	GCGAACTCCCGAACATTTTTAT	A5
301	BARC 3	CGCCTCTTATGGACCAGCCTAT	GCGGTGAGCCATCGGGTTACAAA	A6
302	BARC 8	-	-	1B
303	BARC 24	CAGCGCTCCCCGACTCAGATCCTT	GCGCCATGTTTCTTTTATTACTCACT	B6
304	BARC 37	GCCGCCTACCACAGAGTTGCAGC	GCGGCATTGACAAGACCATAGC	A6
305	BARC 40	CCCAGATGCAATGAAACCACAAT	GCGTAGAACTGAAGCGTAAAATT	A5
306	BARC 45	GCGAGCTGCAGAGGTCCATC	GCGTTAGTCTTCTTGGTCAATCAC	A3
307	BARC 48	GCGAACAGGAG	GCGCTTTCCCACGTTCCATGTTTC	A6-B6-
308	BARC 54	GCGTTGGCTAATCATCGTTCCTTC	AGCACCCTACCCAGCGTCAGTCAA	D6-A3
309	BARC 59	GCG GAG TCT GCA AT	GCA TCC ACC TCC GCA GTC AGT	B5-D2
310	BARC 64	CGCGATCGATCTCCCGGTTTGCT	GGGAAGAGGACCAAGGCCACTA	A7
311	BARC 66	GCGGCATTTACATTTCAGATAGA	TGTGCCTGATTGTAGTAACGTATGT	D1-B2-D7
312	BARC 67	GCGAAAAACGATGCGACTCAAAG	GCGCCATATAATTCAGACCCACAAA	A3
313	BARC 70	GCGTGTCGTGCTTGTTCTCGGTTC	CGCTATTTGCCGCCACCTCCATCA	A4-A7-D7
314	BARC 73	AGGGTTACAGTTTGCTCTTTTAC	CCCGACGACCTATCTATACTTCTCT	B3
315	BARC 75	GCGTATTCTCCCTCGTTTCCAAGT	GTGGGAATTTCTTGGGAGTCTGTA	B3
316	BARC 77	5' CTCCCCGGTCAAGTTTAATCTCT	GCGACATGGGAATTTCAGAAGTGCC	B3
317	BARC 78	GCGAATTAGCATCTGCATCTGTTT	CGGTCAACCAACTACTGCACAAC	A4
318	BARC 80	GCTCACCGGGCATTGGGATCA	GCGATGACGAGATAAAGGTGGAG	B1
319	BARC 87	AGGCCTAGAGACTCAAAGCTG	GCGCTCTTCATCAACACATTCCTC	B3-D7
320	BARC 88	GGGCGCGGCACCAGCACTACC	GGGCGCGGCACCAGCACTACC 3'	B5
321	BARC 89	GCGGTTGTGATGTGCTGAAAGAT	GCGTGGGCTGTTTCTTCCTTTTGTT	B5
322	BARC 92	CGAAGAGACCATTGTATTGAGAA	GCGCATCATAGAGGGGTTGTTCAT	B3-A5
323	BARC 94	GGGGTGTGGTTGTTTGTAAGG	TGCGAATTCTATATACGATCTTGA	B7-A5
324	BARC 95	GGGGTGTGGTTGTTTGTAAGG	TGCGAATTCTATATACGATCTTGA	B7-D2

325	BARC 100	CCGTTAAGCCGCCTACCACAGAG	GGCTTAAAACGGATCCTCCAGGT	A5-A2
326	BARC 113	TCATGCGTGCTAAGTGCTAA	GAGGGCAGGAAAAAGTGACT	A6-A3
327	BARC 117	TCATGCGTGCTAAGTGCTAA	GAGGGCAGGAAAAAGTGACT	A5
328	BARC 128	GCGGGTAGCATTTATGTTGA	CAAACCAGGCAAGAGTCTGA	B1-B2
329	BARC 130	CGGCTAGTAGTTGGAGTGTTGG	ACCGCCTCTAGTTATTGCTCTC	D5
330	BARC 134	CCGTGCTGCAAATGAACAC	AGTTGCCGGTTCCCATTGTCA	B6
331	BARC 135	ATC GCC ATC TCC TCT ACC A	GCG AAC CCA TGT GCT AAG T	A5
332	BARC 140	CGCCAACACCTACCATT	TTCTCCGCACTCACAAAC	B1-B2
333	BARC 141	GGCCCATGGATAATTTTTGAAATG	CAATTCGGCCAAAGAAGAAGTCA	D5
334	BARC 151	TGAGGAAAATGTCTCTATAGCATC	CGCATAAACACCTTCGCTCTTCCA	D3
335	BARC 152	CTTCCTAAAATCGGGCAACCGCTTGT TG	GCGTAATGATGGGAGTGGCTATAGGG CAGTT	B5-D5-B2
336	BARC 165	GCGTAGAGCGGCTGTTAGTGTCAAAT	GCGTTATCTCAAGTTTTGTAGCAG	A5
337	BARC 169	CCGCGAACCATACAAAGGAAAC	GCTATAGAGGCGCCTTGGAGTAC	A5-A7
338	BARC 170	CGCTTGACTTTGAATGGCTGAACA	CGCCCACTTTTTACCTAATCCTTTT	B1- D1
339	BARC 175	GCGTAACAGAAGCGGAGAAAGC	GCGAATCATTTAGTGTTAGGTGGC	A5-A6
340	BARC 178	GCGTATTAGCAAAACAGAAGTGA	GCGACTAGTACGAACACCACAAA	D1 - B6
341	BARC 180	GCGATGCTTGTTTGTTACTTCTC	GCGATGGAACTTCTTTTTGCTCTA	A4
342	BARC 135	ATC GCC ATC TCC TCT ACC A	GCG AAC CCA TGT GCT AAG T	D6
343	BARC 140	CGCCAACACCTACCATT	TTCTCCGCACTCACAAAC	B6
344	BARC 141	GGCCCATGGATAATTTTTGAAATG	CAATTCGGCCAAAGAAGAAGTCA	A5-B3-
345	BARC 151	TGAGGAAAATGTCTCTATAGCATC	CGCATAAACACCTTCGCTCTTCCA	A5-A7
346	BARC 152	CTTCCTAAAATCGGGCAACCGCTTGT	GCGTAATGATGGGAGTGGCTATA	B1- D1
347	BARC 165	GCGTAGAGCGGCTGTTAGTGTCA	GCGTTATCTCAAGTTTTGTAGCAG	A5-A6
348	BARC 169	TGAGGAAAATGTCTCTATAGCATC	CGCATAAACACCTTCGCTCTTCCA	A5-A7
349	BARC 170	CGCTTGACTTTGAATGGCTGAACA	CGCCCACTTTTTACCTAATCCTTTT	A4
350	BARC 175	GCGTAACAGAAGCGGAGAAAGC	GCGAATCATTTAGTGTTAGGTGGC	D6
351	BARC 178	GCGTATTAGCAAAACAGAAGTGA	GCGACTAGTACGAACACCACAAA	B6
352	BARC 180	GCGATGCTTGTTTGTTACTTCTC	GCGATGGAACTTCTTTTTGCTCTA	A5-B3
353	BARC 186	GGAGTGTCGAGATGATGTGGAAA	GCAGACGTCAGCAGCTCGAGAG	A5
354	BARC 197	CGCATGGTCAGTTTTCTTTTAATC	GCGCTCTCCTTCATTTATGGTTTG	A3-A5
355	BARC 204	CGCAGAAGAAAAACCTCGCAGAAAA	CGCAGTGTATCCAAATGGGCAAG	A6 - D6
356	BARC 205	GCGACAGTTGTAGCGGCAGTAGC	GAGCGTAGTAGAAGCAGAAGGAG	D5
357	BARC 206	GCTTTGCCAGGTGAGCACTCT	TGGCCGGGTATTTGAGTTGGAGTT	A4-B3
358	BARC 230	CCC CTC CTC CTT CTC CCT CCT CCT	GGC TCA TGC GGG CGT GTT TGG	A5-B2
359	BARC 240	AGAGGACGCTGAGAACTTTAGAGAA	GCGATCTTTGTAATGCATGGTGAA	A1-B1
360	BARC 255	GTGGCGGCTTGCGGGTGGCTGAGTA	GGGTCGGCTAGCCTTCTTTTATGT	B7
361	BARC 286	GCG AAG AAA ACA TTA GAC CAA	GCG ATA TGT TTC CCG ACA ACT	D5
362	BARC 303	GCG AGC TAT GAT CTG ATG AGG AG	GCG TGT CCT ACT AAT CCA ACT	A5
363	BARC 308	GCG ATC TTG CGT GTG CGT AGG A	GCG TGG GAT GCA AGT GAA CAA	D4-B5
364	BARC 315	CAT CCA GGC GGG CGC ACG AGA	CAA GCC TCC GTG CAC ACC GTA	A4-B7

365	BARC 319	GCA GAG CTA CGG CAA TGT	GCG TAA GTC CCG GAA GTA ACA	A5
366	BARC 320	CGT CTT CAT CAA ATC CGA ACT G	AAA ATC TAT GCG CAG GAG AAA	D5
367	BARC 330	GCA CTA AGC GCT CTT TAT TTA C	CCT GCA TCT GGT ATG GAG A	A5
368	BARC 347	GCG CAC CTC TCC TCA CCT TCT	GCG AAC ATG GAA ATG AAA ACT	D5
369	BARC 352	CCC TTT CTC GCT CGC CTA TCC C	CTG TTT CGC CCA ATC TCG GTG	D7
370	BARC 215	CAC TGA CCT GGT AGC TTG CTC TTT	AGC CAT AGT CTT GAT CTT CGT	A3-B7
371	BARC 216	CGCAGAAGAAAAACCTCGCAGAAAA	CGCAGTGTATCCAAATGGGCAAG	A6 - D6
372	BARC 186	GCGACAGTTGTAGCGGCAGTAGC	GAGCGTAGTAGAAGCAGAAGGAG	D5
373	BARC 197	GCTTTGCCAGGTGAGCACTCT	TGGCCGGGTATTTGAGTTGGAGTT	A4-B3-
374	BARC 204	CCC CTC CTC CTT CTC CCT CCT	CGCAGTGTATCCAC	A5-B2
375	BARC 205	AGAGGACGCTGAGAACTTTAGAG	GCGATCTTTGTAATGCATGGTGAA	A1-B1
376	BARC 206	GTGGCGGCTTGCGGGTGGCTGAG	GGGTCGGCTAGCCTTCTTTTATGT	B7
377	BARC 230	GCG AAG AAA ACA TTA GAC CAA	GCG ATA TGT TTC CCG ACA ACT	D5
378	BARC 240	GCG AGC TAT GAT CTG ATG AGG	GCG TGT CCT ACT AAT CCA ACT	A5
379	BARC 255	GCG ATC TTG CGT GTG CGT AGG	GCG TGG GAT GCA AGT GAA CAA	D4-B5
380	BARC 286	CAT CCA GGC GGG CGC ACG AGA	CAA GCC TCC GTG CAC ACC GTA	A4-B7
381	BARC 303	GCA GAG CTA CGG CAA TGT	' GCG TAA GTC CCG GAA GTA	A5
382	BARC 308	CGT CTT CAT CAA ATC CGA ACT	AAA ATC TAT GCG CAG GAG AAA	D5
383	BARC 315	GCA CTA AGC GCT CTT TAT TTA	CCT GCA TCT GGT ATG GAG A	A5
384	BARC 319	GCG CAC CTC TCC TCA CCT TCT	GCG AAC ATG GAA ATG AAA ACT	D5
385	BARC 320	CCC TTT CTC GCT CGC CTA TCC C	CTG TTT CGC CCA ATC TCG GTG	D7
386	BARC 330	CAC TGA CCT GGT AGC TTG CTC TTT	AGC CAT AGT CTT GAT CTT CGT	A3-B7
387	BARC 347	CGCAGAAGAAAAACCTCGCAGAAAA	CGCAGTGTATCCAAATGGGCAAG	A6 - D6
388	BARC 352	GCGACAGTTGTAGCGGCAGTAGC	GAGCGTAGTAGAAGCAGAAGGAG	D5
389	BARC 215	GCTTTGCCAGGTGAGCACTCT	TGGCCGGGTATTTGAGTTGGAGTT	A4-B3
390	BARC 216	CCC CTC CTC CTT CTC CCT CCT CCT	GGC TCA TGC GGG CGT GTT	A5-B2
400	CFA 2163	TTGATCCTTGATGGGAGGAG	CATCATTGTGTTTACGTTCTTTC	A5
401	CFA 2174	' ACGGCATCACAGGTTAAAGG	GGTCTTTGCACTGCTAGCCT	A7,B7
402	CFA 2185	TTCTTCAGTTGTTTTGGGGG	TTTGGTCGACAAGCAAATCA	A5
403	CFA 2187	TAGCAAAGGGTGCATGTGAG	GCATGTTACGTCGCTGTTGT	A5,D1
404	CFA 2190	CAGTCTGCAATCCACTTTGC	AAAAGGAAACTAAAGCGATG	A5
405	CFA 2234	AATCTGACCGAACAAAATCAC	TCGGAGAGTATTAGAACAGTG	A3
406	CFA 2240	TGCAGCATGCATTTTAGCTT	TGCCGCACTTATTTGTTCAC	A7
407	WMS 2	CTGCAAGCCTGTGATCAACT	CATTCTCAAATGATCGAACA	A3-D3
408	WMS 5	GCCAGCTACCTCGATACAACTC	AGAAAGGGCCAGGCTAGTAGT	A3
409	WMS 6	CGTATCACCTCCTAGCTAAACTAG	AGCCTTATCATGACCCTACCTT	A5-B4
410	WMS 10	CGCACCATCTGTATCATTCTG	TGGTCGTACCAAAGTATACGG	A2-B2
411	WMS 16	GCTTGGACTAGCTAGAGTATCATA	CAATCTTCAATTCTGTCGCACGG	B2-D5
412	WMS 24	CACACAAGGCACCATTGC	CAATGGACATAGTTGTGTGCG	D2-B1
413	WMS 30	ATCTTAGCATAGAAGGGAGTGGG	TTCTGCACCCTGGGTGAT	A2- A4
414	WMS 32	TATGCCGAATTTGTGGACAA	TGCTTGGTCTTGAGCATCAC	A3

415	WMS 33	GGAGTCACACTTGTTTGTGCA	CACTGCACACCTAACTACCTGC	A1-B1
416	WMS 37	ACTTCATTGTTGATCTTGCATG	CGACGAATTCCCAGCTAAAC	D7- D2
417	WMS 43	CACCGACGGTTTCCCTAGAGT	GGTGAGTGCAAATGTCATGTG	B7-A4
418	WMS 44	GTTGAGCTTTTCAGTTCGGC	ACTGGCATCCACTGAGCTG	D7-A4
419	WMS 46	GCACGTGAATGGATTGGAC	TGACCCAATAGTGGTGGTCA	B7
420	WMS 47	TTGCTACCATGCATGACCAT	TTCACCTCGATTGAGGTCCT	B2-A2
421	WMS 52	CTATGAGGCGGAGGTTGAAG	TGCGGTGCTCTTCCATTT	D3
422	WMS 55	GCATCTGGTACACTAGCTGCC	TCATGGATGCATCACATCCT	B2-D6
423	WMS 58	TCTGATCCCGTGAGTGTAACA	GAAAAAAATTGCATATGAGCCC	B6
424	WMS 63	TCGACCTGATCGCCCCTA	CGCCCTGGGTGATGAATAGT	A7
425	WMS 67	ACCACACAAACAAGGTAAGCG	CAACCCTCTTAATTTTGTTGGG	A3-B5
